# 

**Published:** 1842-02

**Authors:** 


### MEDICINE AND SURGERY.

### EDITORS.

---

DANIEL DRAKE, M. D[illegible]

PROFESSOR OF PATHOLOGICAL ANATOMY AND CL[illegible]ICINE

IN THE MEDICAL INSTITUTE OF LOUISVILLE;

LUNSFORD P. YANDELL, M. D.,

PROFESSOR OF CHEMISTRY AND PHARMACY IN THE SAME:

THOMAS W. COLESCOTT, M. D.

### RECEIPTS

### *For the Medical Journal for January,* 1842.

| | |
|---|---|
| Dr. W. M. Dodson, Waynesville, Mo., | $5 00 |
| D. M. Foster, Oak Grove, Ky., | 5 00 |
| R. S. Newton, Gallipolis, Ohio, | 5 00 |
| T. Seaman, Savannah, Tenn., | 5 00 |
| G. D. Morrow, Combs' Store, Tenn. | 5 00 |
| J. Alexander, Dixon's Springs, Tenn., | 10 00 |
| J. T. Wilson, Alton Hill, Tenn. | 5 00 |
| J. C. Helm, Granville. Ia., | 5 00 |
| A. Adams, North Middletown, Ky., | 5 00 |
| W. Anderson, Versailles, Ia., | 5 00 |
| H. M. Witherspoon, Fredonia, Ky., | 5 00 |
| S. Barbour, Milroy, Ia., | 5 00 |
| J. M. Smyth, Green Hill, Tenn., | 10 00 |
| W. W. Walters, Belmont, Ohio, | 5 00 |
| D. Keller, Tuscumbia, Ala., | 5 00 |
| W. M. Mixon, Spring Hill, Miss.. | 5 00 |
| L. Blackwell, Lexington, Mo. | 5 00 |
| C. A. Osman, Millerstown, Ky., | 5 50 |
| M. Hoag, Leroy, Ohio, | 10 00 |
| J. Wood, Clitty, Ia., | 5 00 |
| L. Barton, Waterford, Pa., | 5 00 |
| E. T. Brown, Logan, Ohio, | 5 00 |
| J. H. McFarland, Lebanon, Tenn., | 5 00 |
| W. C. Galt, City, | 5 00 |
| Gale, Vernon, Ia., | 5 00 |
| Spurrier, City, | 5 00 |
| Grady, Hopkinsville, Ky., | 5 00 |
| Kerrick, County, | 5 00 |
| Beasley, Ripley, Ohio, | 5 00 |
| Neblett, Clarksville, Tenn. | 5 00 |
| Shannon, South Union, Ky., | 5 00 |
| J. Gore, Hodgensville, Ky., | 5 00 |
| W. E. Russell, Cadiz, Ky., | 5 00 |
| B. Rogers, Moreland, Ky., | 5 00 |
| V. Payton, City, | 5 00 |
| M. Marsh, Orland, Ia., | 5 00 |
| B. Mullins, Burnt Tavern, Ky., | 5 00 |
| J. W. French, N. Amsterdam, Ia., | 5 00 |
| A. Clapp, N. Albany, Ia., | 5 00 |
| E. W. Eckler, Brownsville, Miss., | 5 00 |

The Western Journal of Medicine and Surgery is published monthly by the undersigned, at the corner of Main and Fifth streets, Louisville, at $5 per annum, payable in advance. Each number contains from 80 to 84 pages making two volumes in the year of about 500 pages.

Contributions to its pages by the Physicians of the Valley of the Mississippi, addressed to the Editors, are respectfully solicited.

Letters on business, to be addressed, postage paid, to the publishers.

☞Postmasters, by the regulations of the Postoffice Department, will frank letters containing subscription money, and all remittances so franked are at the risk of the publishers.

*July* 25, 1841. PRENTICE & WEISSINGER.

